# Uncovering the Genetic Basis of Congenital Heart Disease: Recent Advancements and Implications for Clinical Management

**DOI:** 10.1016/j.cjcpc.2023.10.008

**Published:** 2023-10-19

**Authors:** Karanjot Chhatwal, Jacob J. Smith, Harroop Bola, Abeer Zahid, Ashwin Venkatakrishnan, Thomas Brand

**Affiliations:** a Imperial College School of Medicine, Imperial College London, London, United Kingdom; b National Heart and Lung Institute, Imperial College London, Imperial Center of Clinical and Translational Medicine, London, United Kingdom

## Abstract

Congenital heart disease (CHD) is the most prevalent hereditary disorder, affecting approximately 1% of all live births. A reduction in morbidity and mortality has been achieved with advancements in surgical intervention, yet challenges in managing complications, extracardiac abnormalities, and comorbidities still exist. To address these, a more comprehensive understanding of the genetic basis underlying CHD is required to establish how certain variants are associated with the clinical outcomes. This will enable clinicians to provide personalized treatments by predicting the risk and prognosis, which might improve the therapeutic results and the patient’s quality of life. We review how advancements in genome sequencing are changing our understanding of the genetic basis of CHD, discuss experimental approaches to determine the significance of novel variants, and identify barriers to use this knowledge in the clinics. Next-generation sequencing technologies are unravelling the role of oligogenic inheritance, epigenetic modification, genetic mosaicism, and noncoding variants in controlling the expression of candidate CHD-associated genes. However, clinical risk prediction based on these factors remains challenging. Therefore, studies involving human-induced pluripotent stem cells and single-cell sequencing help create preclinical frameworks for determining the significance of novel genetic variants. Clinicians should be aware of the benefits and implications of the responsible use of genomics. To facilitate and accelerate the clinical integration of these novel technologies, clinicians should actively engage in the latest scientific and technical developments to provide better, more personalized management plans for patients.

## Résumé

La cardiopathie congénitale (CC) est l’affection héréditaire la plus commune, soit environ 1 naissance vivante sur 100. Grâce aux progrès réalisés en chirurgie cardiaque, il a été possible de réduire la morbidité et la mortalité associées à la CC, mais les complications, les anomalies extracardiaques et les affections concomitantes demeurent préoccupantes. Dans ce contexte, il est nécessaire de mieux comprendre les fondements génétiques de la CC pour déterminer les variants qui sont à l’origine des complications cliniques. Les cliniciens pourront ainsi proposer des traitements personnalisés en tenant compte du risque et du pronostic dans l’espoir d’améliorer les résultats thérapeutiques et la qualité de vie des patients. Nous revenons ici sur les avancées réalisées dans le séquençage du génome et sur la façon dont elles ont changé notre compréhension des fondements génétiques de la CC. Nous décrivons les techniques expérimentales utilisées pour mettre au jour de nouveaux variants d’intérêt et présentons les obstacles qui empêchent d’utiliser ces connaissances en clinique. Les techniques de séquençage de nouvelle génération permettent de lever le voile sur le rôle de la transmission oligogénique, de la modification épigénétique, de la mosaïque génétique et des variants non codants dans la régulation de l’expression des gènes candidats associés à la CC. La prédiction du risque clinique en fonction de ces facteurs demeure toutefois hasardeuse. Les études sur les cellules souches pluripotentes induites et le séquençage unicellulaire aident à établir le cadre préclinique nécessaire pour déterminer l’importance des nouveaux variants génétiques. Les cliniciens doivent être conscients des bienfaits et de la portée que peut avoir la génomique lorsqu’elle est utilisée de façon responsable. Pour faciliter et accélérer l’intégration clinique de ces nouvelles technologies, les cliniciens doivent s’intéresser de près aux derniers développements scientifiques et techniques s’ils veulent un jour proposer à leurs patients un plan de traitement plus pertinent et plus personnalisé.

Congenital heart disease (CHD) is the most common birth defect, and cardiac malformations affect approximately 1% of all live births.^1^^,^^2^ Approximately 12 million people globally live with confirmed CHD.^3^ CHD theoretically refers to any inherited cardiac disease, spanning structural heart disease, hereditary arrhythmias, and cardiomyopathies. For this review, we restricted the definition of CHD to structural abnormalities of the heart or great vessels present at birth.^4^

Over the last 70 years, surgical advancements, the mainstay of CHD treatment, have led to a 75% increase in newborns with CHD surviving into adulthood and a 34.5% reduction in infant (<1 year) CHD-related deaths between 1990 and 2017.^3^^,^^5^ Surgical management can correct significant structural abnormalities; however, patients still have a 17-fold increased mortality risk and an elevated risk of developing multiple comorbidities.^6^

CHD inheritance is complex. Large structural abnormalities can develop early in embryonic development and affect the development of multiple organ systems. Prognosis varies based on underlying structural abnormalities, and 13% of newborn babies have extracardiac abnormalities or functional defects, which can lead to associated neurodevelopmental delay. Genetic variants are implicated in 34% of CHD cases as the primary driver of pathogenesis, but genetic penetrance of variants to phenotype and clinical course varies significantly. Thus, decoding the complex gene regulatory networks that have evolved around these genes is critical to deciphering phenotype heterogeneity. Advancements in sequencing technologies such as gene panel testing (GPT) and whole-genome sequencing (WGS) and improvements in understanding the mechanistic sequelae of CHD will allow increasingly personalized management based on an individual’s risk profile.

In this review, we first focus on specific genes pivotal to cardiac development, how genomic technology is helping to decipher their complex regulatory networks, and the use of different experimental models to define the pathomechanisms involved. We then discuss the methods of available genetic testing and implications of their incorporation into clinical practice.

We aim to provide a clinically relevant overview of the recent advancements in knowledge of CHD etiology, discuss how genetic testing is changing clinical practice, and point to the increasing role of artificial intelligence (AI).

## Genetic Control of Cardiogenesis

This section will discuss the role of key genes frequently implicated in CHD in cardiogenesis and the developmental implications of their aberrant expression. Cardiac development is a tightly controlled process choreographed by cardiac transcription factors (cTFs). Spatiotemporal expression of key genes and regulators determining cardiac cell lineage drives the development and differentiation of the cardiovascular system.^7^ During this time, the heart is most prone to errors and small aberrations in gene expression, or dosage can cause malformation in heart structures, leading to CHD.^8^

Approximately 400 genes have already been implicated in CHD, including those encoding (1) important and interconnected cTFs governing cardiac development (NKX2.5, GATA4, and members of the T-box family [TBX1 and TBX5]); (2) structural proteins (MYH6, ACTC1, and ELN); and (3) signal factors (neurogenic locus notch homolog protein 1 and vascular endothelial growth factor).^9^, ^10^, ^11^, ^12^ Although mutations in the coding sequence or gene regulatory sequences affecting their expression can result in cardiac malformations, the precise genotype/phenotype relationship is often challenging to establish.^13^^,^^14^

### NKX2.5

NKX2.5 is the master regulator of cardiac development. Ectopic expression of *nkx2.5* in zebrafish expands the cardiogenic field.^15^ Mice carrying a loss-of-function (LOF) *Nkx2.5* mutation can initiate cardiogenesis but die due to improper cardiac looping.^16^ Heart development is initiated but arrested early, indicating a degree of inbuilt redundancy among the members of the *NK2* gene family in mammals. Mutations in *NKX2.5* are often associated with atrial septal defects (ASDs) and conduction abnormalities. *NKX2.5* mutations have also been reported in ventricular septal defect (VSD), tetralogy of Fallot (TOF), aortic stenosis, and hypoplastic left heart syndrome (HLHS), demonstrating that NKX2.5 has multiple roles during heart development.^17^, ^18^, ^19^, ^20^, ^21^

### GATA4

GATA4 is a potent activator of many cardiac genes, including the genes encoding natriuretic peptides (*NPPA* and *NPPB*), cardiac myosin heavy chain (*MYH6* and *MYH7*), and troponin isoforms (*TNNI3* and *TNNC1*) as well as the cardiac muscarinic m2 acetylcholine receptor (CHRM2). *GATA4* is a mutual cofactor of *NKX2.5*; neither alone is sufficient to initiate cardiogenesis.^22^, ^23^, ^24^, ^25^ *GATA4* and the related *GATA5* and *GATA6* are expressed within the developing heart, and variants of GATA genes have been implicated in CHD.^18^ *GATA4* variants have been associated with ASD, VSD, and TOF.^26^

### *TBX1* and *TBX5*

The T-box family of transcription factors plays an important role in patterning the embryonic germ layers.^27^ *TBX5* is highly expressed in the forelimb buds and the developing heart, whereas *TBX1* is expressed in pharyngeal endoderm, mesoderm, and ectoderm. LOF mutations in *TBX1* or *TBX5* result in the dramatic cardiovascular phenotypes seen in 22q11.2 deletion syndrome and Holt-Oram syndrome, respectively.^28^^,^^29^ Isolated variants in *TBX1* produce highly variable phenotypes. Overexpression of *TBX1* in mice results in an aberrant right subclavian artery, TOF, with atresia of the main pulmonary arteries, and abnormalities in the left ductus arteriosus, whereas heterozygous null mutations in mice can result in atresia of the fourth aortic arch artery and persistence of the distal right dorsal aorta.^30^, ^31^, ^32^ In humans, variants in *TBX1* are commonly associated with abnormalities in pharyngeal arch patterning and ventricular septation. Evidently, the role of *TBX1* is complex and dose dependent; thus, small variants, including single nucleotide polymorphisms (SNPs) in *TBX1* or variations in its expression level, can have diverse consequences.^30^, ^31^, ^32^

Lindsay et al.^33^ used heterozygous *Tbx1* null mutant mice and demonstrated that haploinsufficiency results in the characteristic cardiopharyngeal phenotype, as seen in 22q11.2 deletion syndrome. Zebrafish *wnt-11r* and alcama have been shown to function downstream of *tbx1, wnt11r* knockout mutants display similar looping and patterning defects as *tbx1* knockout mutants and forced expression of alcama, and *wnt11r* partially rescued the cardiac defects.^34^ It is hoped that identifying gene regulatory networks coupled with further studies using human-induced pluripotent stem cells (hiPSCs) may lead to identifying new therapeutics.

## Genetic Basis of CHD

**Figure 1:**
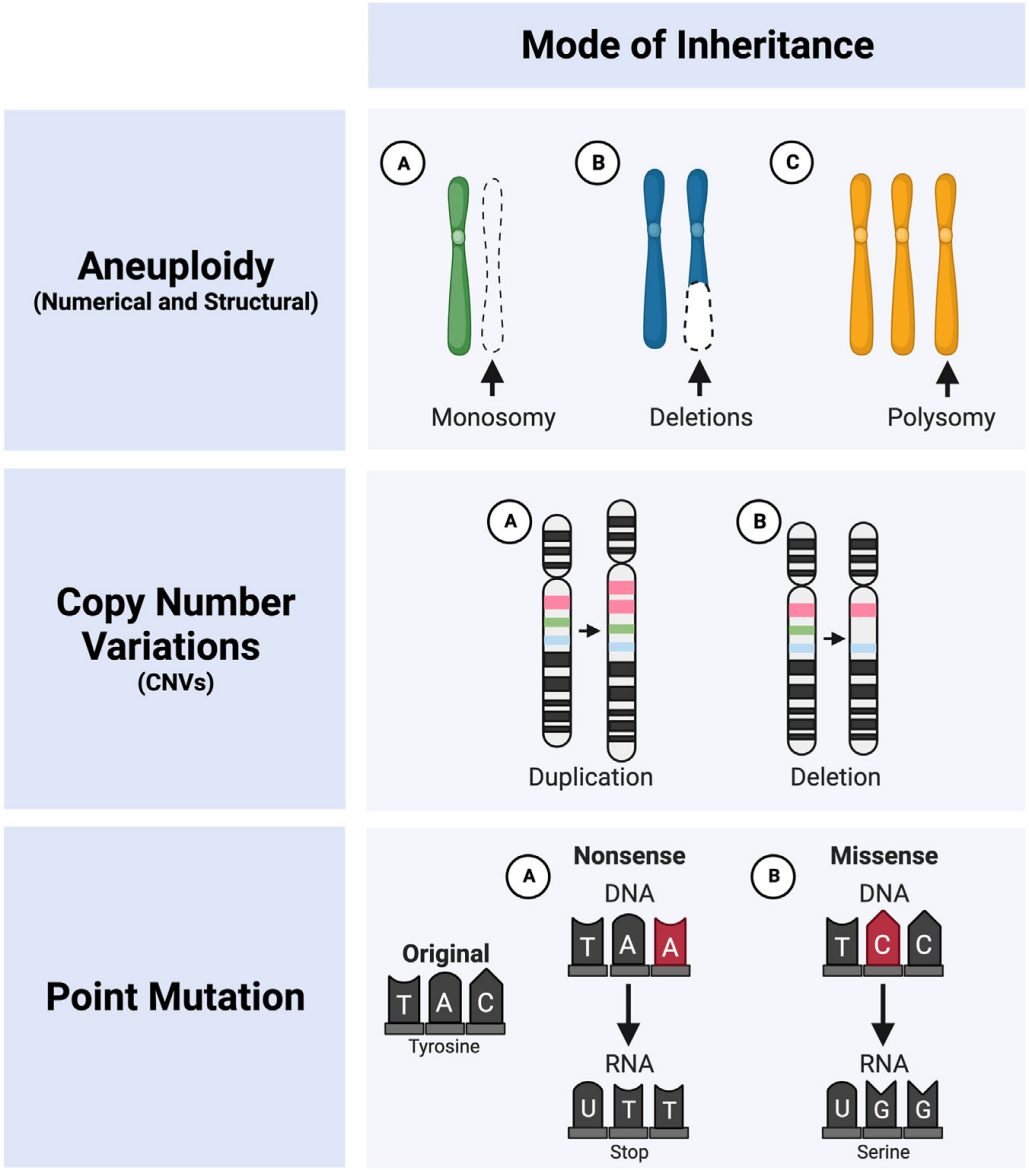
Mode of inheritance. Genetic aberrations, including aneuploidy, copy number variations, and point mutations, contribute to the pathogenesis of congenital heart disease, as illustrated. This figure has been created using BioRender.

Although the etiology of CHD is multifactorial, recent advancements in genetics have unveiled a profound role for genomic variations in its pathogenesis. Aneuploidies characterized by chromosomal aberrations and copy number variations (CNVs) involving structural genomic imbalances have been identified as critical contributors to the genetic basis of CHD (The definitions of the genetic changes are summarized in Box 1). In addition, monogenic inheritance patterns involving mutations in single genes have been increasingly recognized as causative factors in a subset of CHD cases. Each of these types of genetic alterations and their respective mechanism of inheritance are depicted in Figure 1. Therefore, understanding the intricate interplay between these genetic alterations is essential to appreciate the genetic underpinnings of CHD better and thereby facilitate earlier diagnosis, prognostication, and personalized therapeutic strategies.

Box 1 **List of genetic terms**

**Table:** 

*Aneuploidy* A change in chromosome number, which could either be an increase (polysomy), a reduction by half (monsomy), or a complete loss (nullisomy) of a chromosome. *Copy number variations (CNVs)* A genetic phenomenon where sequences in the genome are repeated or deleted, and the copy number varies between individuals. *De novo variants (DNVs)* Genomic variants that are not inherited from a parent but which have arisen in an individual for the first time. The formation of a DNV is based on the spontaneous mutation rate during DNA replication or is due to mutagens such as radiation and certain chemicals. *Epigenetics* This is a term that describes changes that do not affect the sequence of genomic DNA but determine how cells in the body access the information on the genomic DNA. *Epigenetic modifiers* Proteins that modify the epigenome through methylation of the genomic DNA, the modification of the chromatin (eg, histone modifications [acetylation, methylation, phosphorylation, etc.]), or structural chromatin modifications (chromatin remodelling). *Genetic variants* This term describes a change in the DNA sequence of a gene. In contrast to the term mutations, variants do not make any presumptions regarding the functional consequences of the sequence variation. Further experiments are required to determine the pathogenic properties of a newly discovered genetic variant. *Variants of unknown significance (VUS)* Variants that have been discovered by genetic testing for which it is presently unclear whether they are causally associated with a pathology are termed VUS. To assess the significance of a newly discovered variant, a prediction by computational methods, an experimental analysis of the variant in cell lines and animal models, and structural analysis may subsequently lead to a reclassification of a variant.

### Aneuploidies

An imbalance in chromosome numbers due to the addition or deletion of a chromosome results in aneuploidy. Subsequent genetic dysregulation manifests with severe pleiotropic effects on cardiac development. Because of the exquisite dose sensitivity of genes coordinating cardiac morphogenesis, the severity of CHDs is thought to be dose dependent; in aneuploidies, particularly trisomy 21, the additional quantity of dosage-sensitive genes results in the overexpression of cTFs, causing aberrant heart development.^35^ Trisomy-18, -21 and monosomy X commonly result in septal and outflow tract defects.^36^, ^37^, ^38^, ^39^, ^40^ Aneuploidies are often also associated with extracardiac abnormalities, known as syndromic CHDs, which account for approximately 8%-13% of total CHDs.^36^, ^37^, ^38^, ^39^, ^40^

A total of 40%-60% of patients with trisomy 21 develop CHD. Most frequently, cardiac malformations include atrioventricular septal defects, VSDs, ASDs, and patent ductus arteriosus.^41^^,^^42^ Prenatal karyotyping is the first line for detecting aneuploidies and chromosomal abnormalities in trisomy 21; however, comparative genomic hybridization (CGH) arrays can detect CNVs that are not recognized by karyotyping.^43^^,^^44^

### Copy number variations

CNVs refer to variations in the number of copies of specific genomic DNA sequences, which are repeated or reduced in number and either inherited or occur *de novo* and represent 10%-15% of all CHD etiologies.^45^ Chromosomal microarray analysis (CMA) is the gold standard for detecting CNVs, and studies have demonstrated the significant advantage CMAs have over karyotyping. Wapner et al.^46^ showed that CMA detected clinically relevant duplications and deletions in 6% of samples with a standard karyotype. Furthermore, Goldmuntz et al.^47^ used (genome-wide) microarray analysis and identified 12 novel rare pathogenic CNVs associated with genes (*RP1, NTRK3, MESP1, ADAM19,* and *HAND1*) known to be important in cardiac development. Similarly, Glessner et al.^48^ used genome-wide arrays and whole-exome sequencing (WES) in CHD trios and found a significantly increased frequency of *de novo* CNVs. This highlights the importance of (genome-wide) microarray analysis to detect previously unrecognized phenotype-altering genetic variations observed in CHD. Moreover, CNV length has been shown to be negatively associated with postsurgical outcomes in chromosomal aneuploidies; hence, clinically, quantification of CNV length can be used as a novel tool in risk stratification.^49^^,^^50^

### The role of monogenic inheritance

WES analysis of Paediatric Cardiac Genomics Consortium (PCGC) patient data has accelerated the identification of CHD candidate genes, revealing specific *de novo* and inherited variants.^12^^,^^51^^,^^52^ Subsequent exome sequencing and gene set enrichment analysis identified novel CHD genes. Edwards et al.^53^ used zebrafish knockout models of genes identified by PCGC to establish a functional relationship between dysregulated genes encoding proteins of the Wave2 complex, which are involved in the control of microfilament organization and left ventricular outflow tract obstruction lesions.

It has been postulated that variants of cTF contribute to gene network dysregulation due to impaired protein-protein or protein-DNA interactions in human CHD. Variants in *GATA4* have been implicated in cardiac septal defects. The functional role of *GATA4* has been studied in hiPSC-derived cardiomyocytes (hiPSC-CM) and cardiac progenitor cell models.^54^, ^55^, ^56^ Ang et al.^54^ analysed the pathogenic effect of a *GATA4 p.G296S* heterozygous missense variant in causing cardiac malformations and demonstrated an impaired interaction of *GATA4* and *TBX5*. RNA-seq analysis of hiPSCs carrying the *GATA4 p.G296S* variant revealed that the expression of genes involved in endocardial cushion formation and septal morphogenesis were dysregulated. These findings demonstrate the utility of hiPSCs as an *in vitro* model to study cellular events contributing to morphogenetic defects.^54^

Gonzalez-Teran et al.^56^ further explored the role of GATA4 and TBX5 interactions using hiPSCs integrated with WES analysis of 9000 proband-parent trios to identify *de novo* missense variants associated with CHD. This analysis identified glyoxylate reductase 1 homolog (*GLYR1*), an epigenetic reader gene that co-occupies cardiac developmental genes and demonstrated that a *de novo* variant (DNV), *P495L*, led to impaired GLYR1-GATA4 interaction and enhanced susceptibility to cardiac malformation in a murine model. The study provides *in vivo* evidence to explain heterogeneity in the resulting cardiac phenotype in presumptive single-gene defect-associated CHDs by revealing the increased penetrance of the disease phenotype in GATA4 haploinsufficient mice in the presence of the *P495L* variant. Evidently, in many cases, monogenic inheritance alone cannot explain the complex genetics of CHD. Additional gene variants can influence the expression and penetrance of monogenic mutations, known as oligogenic inheritance.^56^ Further studies integrating genome-wide association studies and genome linkage analysis are required to assess the effects of synergistic interactions on gene expression.

## Heterogeneity in CHD Phenotypes

Cohort WGS studies have identified rare transmitting variants and DNVs in 8% of patients with sporadic CHD.^12^ Although cohort WGS/WES have detected aneuploidy, CNVs, and insertion-deletion mutations, it is predicted that only 45% of CHD cases are likely to be a result of these aberrations, and there is a lack of a clear genotype-phenotype correlation.^8^

Cardiac development is susceptible to gene dosage effects of critical cTFs; hence, it is plausible that genetic control of gene expression could drastically affect the disease phenotype.^4^ The complexity of the underlying genetics, evidence of gene-environment interactions, and the effect of noncoding RNA all likely also play a role in determining the extent of phenotypic expression of patients with damaging CHD variants.^8^

### *De novo* variants

Until recently, it has been difficult to determine the exact contribution of DNVs in CHD. Around 1% of the human genome consists of gene coding regions, and there is a high level of sequence conservation between species. Mutations of coding regions often display a Mendelian inheritance pattern with a high likelihood of phenotypic consequence, although predicting clinical manifestation is difficult.^4^ WES can now be performed at around 20% of the cost of WGS, and decreasing costs have enabled family genome studies to be performed, identifying DNVs associated with CHD.^4^ DNVs are rare, with an incidence of approximately 1.8 × 10^−8^/nucleotide replication, but have not been subject to evolutionary pressures, so they are more likely to be phenotypically damaging.^57^ Because most cases of CHD are associated with impaired reproductive fitness, the estimated contribution of DNVs to CHD and neurodevelopmental disorders is relatively high. Exome sequencing of 1213 parent-offspring trios revealed that DNVs were responsible for 20% of patients with CHD, neurodevelopmental disorders, and extracardiac abnormalities but accounted for only 2% of patients with cardiac abnormalities alone.^58^ Some disease manifestations of DNVs may present later in life, so early exome profiling of patients predicted to be at risk is important. Analysis of 362 parent-offspring trios from a wider cohort of 5000 probands found a marked excess in DNVs in chromatin remodelling genes (encoding regulators of H3K4 and H3K27 methylation) in severely symptomatic CHD.^9^ H3K4me is an activating mark, and H3K27me is a deactivating mark in gene promoter regions conferring temporal changes in chromatin accessibility, which are critical to embryonic development.^9^

### Epigenetic modifications

Gene expression can also be regulated indirectly (epigenetically) without altering the DNA sequence of any CHD-related gene through changes during transcription, translation, or post-translation. Some mechanisms involve histone modification, chromatin remodelling, and microRNAs (miRNAs).^59^, ^60^, ^61^ Moreover, we will also discuss gene-environment interactions, such as changes in the intrauterine environment and external environmental cues and their role in CHD.^62^

In cells, genomic DNA is coiled around nucleosomes, octameric protein complexes consisting of several different histone subunits.^63^ The higher-order structure of the DNA nucleosome complex determines the accessibility of any gene by transcription factors and, thus, determines whether a gene is transcriptionally active or silent. Chromatin accessibility is regulated through post-translational histone modifications, which can take many forms, such as methylation and acetylation.^64^ In addition, genomic DNA can be directly methylated and thereby silenced as this occurs primarily at the transcriptional start sites of a gene, which is also termed CpG islands because of their high GC content.^65^ Murine cardiomyocytes isolated from fetal and neonatal hearts displayed maturation-dependent differences in their DNA methylation pattern of CpG islands of cardiac development genes.^66^ Grunert et al.^59^ compared the methylation patterns of myocardial biopsy samples of patients with TOF or VSD and found a substantial similarity in the extent of methylation of promoters of genes involved in mRNA splicing of the diseased tissue vs healthy controls.

Significantly, aberrant mRNA splicing is common in CHD: 50% of genes known to be critical to heart development were found to be aberrantly spliced in patients with TOF.^67^

It has been shown that there is a marked excess in DNVs affecting histone-modifying genes in patients with CHD, leading to altered methylation, particularly of genes heavily expressed in the heart.^9^ Changes in histone acetylation have also been associated with CHD. Decreased acetylation of H3K4, H3K9, and H3K27 after the downregulation of the histone acetyltransferase has been linked to CHD through decreased GATA4 expression.^68^ Absent histone deacetylase 2 has been shown to cause severe cardiac developmental defects and myocyte hyperproliferation, possibly through hyperacetylation of GATA4.^69^

Defects in DNA remodelling complexes have also been associated with CHD.^60^^,^^70^, ^71^, ^72^ Chromatin remodelling refers to the process of repositioning, ejecting, and restructuring nucleosomes, thus regulating the accessibility of DNA sequences to the transcription machinery.^64^ Several studies in mice have shown that a deficiency in Brg1 encoding an ATPase subunit of a DNA remodelling complex essential for cardiac development causes congenital heart defects.^60^^,^^70^^,^^73^ More recently, a case-control study found significantly lower levels of BRG1 expression in the myocardium of patients with CHD compared with controls.^71^ Interestingly, GATA4 expression was directly correlated with the BRG1 expression levels in the myocardium of patients with CHD, suggesting that the pathogenic effects of BRG1 deficiency could be due to its impact on GATA4 expression. Mutations in another chromatin remodeller, CHD7, have also been associated with CHD, specifically associated with atrioventricular septal and conotruncal defects.^74^

miRNA is another epigenetic regulator crucial for normal cardiac development. miRNAs inhibit the translation by binding to their target mRNAs.^61^ Typically, miRNAs regulate the expression of multiple transcripts encoding proteins that act together in a regulatory pathway. Both high and low miRNA expression levels have been shown to cause CHD.^61^^,^^75^, ^76^, ^77^ For instance, excess miR-1 miRNA abundantly expressed in the heart suppressed ventricular cardiomyocyte proliferation, while targeted miR-1-2 deletion caused VSD.^61^^,^^78^^,^^79^ The results can be attributed to altered expression levels of miRNA targets such as the transcription factor Hand2 and the histone-modifying protein HDAC4.^61^^,^^78^^,^^79^ Downregulation of miR-206 and miR-240 and upregulation of miR-424/424∗ and miR-421 have all also been linked to TOF by causing changes in the expression of target genes involved in intercellular communication (GJA1), cardiac septation (NF1 and HAS2), and cardiac outflow tract development (JARDI2).^75^, ^76^, ^77^^,^^80^

It is well characterized that epigenetic changes occur because of changes in the intrauterine environment.^81^ Gene dosage and spatiotemporal expression are critical to cardiac development, so the developing heart is susceptible to small changes in the internal and external environment. Environmental cues drive heart development, and haemodynamic flow is vital to the proper anatomic and electrophysiological development of the heart.^82^^,^^83^ A large cohort study found that the risk of CHD is 60% greater among monochorionic and diamniotic twins.^84^ Monochorionicity resulted in a 9-fold increase in the incidence of CHD. Abnormal placentation and, consequently, an abnormal blood flow distribution among the twins can result in twin-twin transfusion syndrome. Alone, this syndrome is associated with a 13-fold increase in the risk of CHD, but specific heart defects vary between twins.^84^ There is an increased incidence of valvular stenosis and hypoplastic ventricles associated with reduced haemodynamic flow in donor twins. The opposite effect is observed where the prevalence of obstructive lesions and valvular regurgitation is increased among recipient twins.^85^^,^^86^ Because both individuals are assumed to be genetically identical, CHD malformations are likely to directly result from epigenetic modifications, environmental factors, or abnormal placentation. Seminal studies in monozygotic twins have revolutionized the understanding of epigenetics, and more studies are needed to determine the exact role of epigenetics in CHD.^87^^,^^88^

It is estimated that environmental cues are responsible for 2%-10% of CHD cases. Maternal illness, smoking, obesity, and alcohol have all been associated with CHD.^89^ Maternal diabetes mellitus increases the risk of CHD 4-fold.^90^ Globally, diabetes mellitus is increasing, so identifying gene regulatory pathways affected by diabetes mellitus is a research priority in reducing the risk of CHD.^91^ In murine models, haploinsufficiency of neurogenic locus notch homolog protein 1 and simultaneous hyperglycaemia or hypoxia increased the incidence of CHD.^92^^,^^93^ Maternal exposure to teratogens such as thalidomide and isotretinoin has also been associated with CHD cases such as ASDs and VSDs.^89^ The list of environmental cues that can potentially cause CHD is nonexhaustive. It is clear though that CHD pathogenesis is complex, and epigenetics play a vital role.

### Mosaicism

Mosaicism is caused by mutational processes occurring during early embryonic development, such as the dividing zygote or early embryo resulting in 2 or more cell populations with a distinct genotype.^8^ Cases of CHD as a result of mosaicism are rare and predicted to account only for approximately 1%-2% of CHD cases.^94^ Mosaicism may result in undetected germline DNV mutations that do not affect the carrier’s phenotype. More research is needed to determine the contribution of such inheritance patterns to CHD.

### Noncoding variants

The ENCODE study, which aimed to determine the biological function of the 3 billion bases of the human genome, identified that noncoding DNA (ncDNA) is associated with regulatory functions governing transcription, chromatin structure, and histone modification.^95^ NcDNA is associated with slight variations in the expression of target genes, so it is difficult to define a threshold over which ncDNA variants are considered damaging. WGS is necessary to explore ncDNA variants in CHD cohorts, but the cost, data processing, and genetic noise are limitations to its current use.^96^ Determining the functional significance of variants in ncDNA is difficult. *In vitro* methods have insufficient power to measure the effects of ncDNA variants, which in isolation do not sufficiently affect gene expression to an extent in which alteration to phenotype is observed. In many cases, ncDNA acts as a “dampening” or “amplifying” signal to gene expression, and so the inheritance of multiple variants simultaneously is required to develop disease.^97^ However, there is much lower sequence conservation between individuals than in coding DNA, and so traditional genome association analysis with defined reference populations is difficult.

### Modelling heart disease—*in vitro* and *in vivo* models to infer causality

Determining the functional significance of genetic variants identified by sequencing technologies such as genome-wide association studies and WGS is challenging. Animal models, hiPSC-CM, and single-cell transcriptomics (SCTs) can be used to determine the association of variants with CHD.^97^

Culture protocols have been developed to differentiate hiPSCs into pacemaker cells, atrial or ventricular cardiomyocytes, and other cell types found in the developing heart.^98^ It is possible before differentiation to subject hiPSCs to CRISPR/Cas9-mediated gene editing generating LOF alleles to establish gene function or to create missense or nonsense alleles to model genetic variants found in patients.^99^, ^100^, ^101^, ^102^, ^103^, ^104^ WES can identify gene variants inherited oligogenically.^105^ Gifford et al.,^100^ for example, identified *NKX2.5* as a modifier in the oligogenic inheritance of left ventricular noncompaction cardiomyopathy using WES. The extent of differentiation of hiPSC-CM is limited, and its maturity level is equal to cardiomyocytes found in the fetal heart; therefore, disease phenotypes, particularly those acquired later in life, may not fully manifest.^106^ More importantly, the 2-dimensional culture conditions that are often used are not able to recapitulate the complex cell-cell interactions that occur during heart development. However, multiple approaches to enhance cardiomyocyte maturation or to make use of 3-dimensional culture models have been developed, such as cardiac organoids, microtissues, engineered heart tissues, and biomimetic culture systems, which may help to improve the suitability of hiPSCs to model CHD in a dish.^106^, ^107^, ^108^, ^109^, ^110^, ^111^, ^112^

In parallel to any cell culture–based validation approach, genetic variants should also be studied using *in vivo* models. Humans and mice share a similar cardiac anatomy and gene expression pattern, and display strong sequence conservation and similar sequelae of cardiogenesis; however, murine models are expensive and differ in cardiac physiology, and LOF mutants are often nonviable, preventing longitudinal studies.^113^ Zebrafish and Drosophila models are cost-effective and simpler to manipulate genetically but display a more primitive heart structure, which often limits their utility in modelling CHD.^113^ Finally, SCTs enable the mapping of cell types within the heart to determine their lineage relationship on basis of their differential gene expression pattern.^114^ SCTs have, for example, identified 4 distinct cell lineages of healthy ventricular cardiomyocytes, catalogued transcriptional signatures of progenitor cells, and have shown that the start of MESP1 expression demarcates the loss of pluripotency in mouse embryos.^115^, ^116^, ^117^ Integrating cell lineage mapping using SCTs with hiPSCs and *in vivo* models will be important to contextualize and determine the functional significance of newly discovered variants.^104^

## Genomic Tools for the Clinical Practice of CHD

Congenital heart defects can occur in isolation of the heart or with multiple congenital abnormalities that can affect multiple organ systems. Single gene “syndromes” such as 22q11.2 deletion syndrome are noted for their heterogeneous presentation and penetrance, resulting in presentations ranging from isolated cardiac abnormalities to clinically overt defects in multiple organ systems such as thymic hypoplasia, abnormalities of the palate, malignancy, renal insufficiency, and neurodevelopmental delay.^118^ In 22q11.2 deletion syndrome, cytogenic or fluorescence *in situ* hybridization (FISH) analysis is routinely performed in individuals of high clinical suspicion or after newborn T-cell receptor excision circle screening.^119^ This is a similar story across clinical practice in CHD; clinically overt cases with a high index of suspicion for single gene defects or CNVs are increasingly subject to genetic diagnosis, and thus, the next sections will detail the currently available genomic tools available for clinic at the time of writing.

### Karyotyping

Karyotyping facilitates the visualization and analysis of chromosomal composition at the macroscopic level to elucidate chromosomal aberrations, including aneuploidies. Clinically, karyotyping has been employed as the preferred prenatal screening test to detect structural abnormalities encompassing large chromosomal segments.^120^ However, G-banding karyotyping is limited to a resolution of 5-10 mb, subsequently being inefficacious in detecting small genomic imbalances and CNVs. As a screening test, the diagnostic detection rate can be enhanced with the addition of subtelomeric FISH to detect submicroscopic deletions and duplications.^120^^,^^121^

### Fluorescence *in situ* hybridization

FISH enables the visualization and subsequent detection of the presence and location of specific DNA sequences on chromosomes and in cell nuclei. Using fluorescently labelled DNA probes that can hybridize to complementary genetic material within a cell, genetic alterations and chromosomal abnormalities associated with CHD can be elucidated.

One such example is 22q11.2 deletion syndrome characterized by a <3 mb microdeletion that is conventionally diagnosed using FISH, mitigating the limitations associated with low-resolution karyotype analysis.^122^ The inability to comprehensively assess the whole genome is limiting for signals outside the region of hybridization by the FISH probe, resulting in false-negative detection in clinically suspected 22q11.2 deletion syndrome.^123^

### Chromosomal microarray analysis and comparative genomic hybridization arrays

Several studies have demonstrated the incremental diagnostic yield of microarray analysis compared with conventional karyotyping.^120^^,^^124^, ^125^, ^126^, ^127^ CMA is a high-resolution genome-wide cytogenetic technique used for the detection of submicroscopic chromosomal imbalances, including CNVs, across the entire genome. CMA predominantly encompasses 2 primary cytogenetic techniques: CGH arrays and SNPs. In the context of noninformative karyotype analysis, high-resolution array CGH analysis has demonstrated an incremental diagnostic yield (approximately 15%-20%) in prenatal CHD screening, conferring additional value in identifying pathogenic CNVs, and detecting variants of unknown significance (VUS).^125^ As such, array CGHs are a recommended first-line diagnostic test in clinically suspected CHD, especially in those with concomitant multiple congenital anomalies, as opposed to patients with detectable common aneuploidy syndromes such as trisomy 13—owing to the high sensitivity for submicroscopic duplications and deletions in CMA.^46^^,^^120^^,^^125^

**TABLE 1:** Genetic testing indications

Test	Definition	Associated CHD conditions	References
Fluorescence *in situ* hybridization	Detection of suspected deletion or duplication syndromes of specific DNA regions	Trisomy 21, -18, and -13 Turner syndrome Williams syndrome 22q11.2 deletion syndrome	^136^^,^^137^
Multiplex ligation-dependent probe amplification	Assessment of known microdeletion/duplication syndromes. Detection of CNVs	1p36 deletion syndrome Williams syndrome 22q11.2 deletion syndrome	^137^^,^^138^
Chromosomal microarray analysis	Evaluation of patients with multiple congenital anomalies to identify underlying chromosomal abnormalities or CNVs affecting critical periods of cardiac development	HLHS CHARGE syndrome Jacobsen syndrome Alagille syndrome 22q11.2 deletion syndrome	^129^^,^^137^^,^^139^
Whole-exome sequencing	Selective sequencing of protein-coding regions, accounting for approximately 1% of the genome. Precise identification of single-nucleotide variants, CNVs, insertions, and microdeletions	TOF ASD PDA HLHS Aortic valve stenosis	140, 141, 142
Whole-genome sequencing	Sequencing of the entire genome, including noncoding and protein-encoding regions. Identification of SNVs, insertions, microdeletions, and structural variants	TOF Coarctation of the aorta (CoA) CHARGE syndrome Alagille syndrome	^8^^,^^143^
Comparative genomic hybridization arrays	Assessment of unbalanced large CNV changes and genomic rearrangements	22q11.2 deletion syndrome Wolf-Hirschhorn syndrome Miller-Dieker syndrome HLHS TOF VSD CoA	^129^

ASD, atrial septal defect; CHARGE, coloboma, heart defects, atresia choanae, growth retardation, genital and ear abnormalities; CHD, congenital heart disease; CNV, copy number variation; HLHS, hypoplastic left heart syndrome; PDA, patent ductus arteriosus; SNV, single nucleotide variant; TOF, tetralogy of Fallot; VSD, ventricular septal defect.

CGH arrays are limited as they cannot isolate structural variations or identify the specific genetic locus implicated in CHD.^128^, ^129^, ^130^ Furthermore, studies have demonstrated the importance of epigenetic changes and ncDNA for the development of CHD, which are not identified by CGH arrays.^59^, ^131^, ^132^, ^133^, ^134^ Advancements in WGS could address some of the limitations of CGH arrays.^135^ (The indications for genetic testing are summarized in Table 1 and their respective technical descriptions in Box 2).

**Box 2 Molecular genetic techniques in congenital heart disease (CHD) diagnostic**

**Table:** 

*CHD trios* Trio analysis performs a whole-exome or clinical exome sequencing analysis of the parents and the offspring who have developed CHD. Trio analysis will identify genetic alterations such as insertions or deletions (indels), single nucleotide variants, and copy number variations (CNVs). Because the parents do not have the child’s disease, this comprehensive analysis reduces false-positive calls and enables the prioritization of potential disease-causing variants. *Sequencin*g *techniques: whole-exome sequencing (WES), whole-genome sequencing (WGS), and single-cell transcriptomics* Sequencing technology, large data handling, and bioinformatic analysis have significantly improved in recent years. Thus, the entire protein encoding transcriptome can be sequenced as in WES. A more targeted approach of clinical exome sequencing is trying to reduce the amount of data analysis by sequencing a comprehensive list of genes, which have been validated and are therefore known to be associated with a particular group of diseases such as the CHD comprehensive gene panel introduced in Table II. To identify variants affecting the gene regulatory elements and thereby causing an increase or decrease in gene expression, the entire genome is sequenced in WGS. Another recently introduced approach is called single-cell sequencing, which assesses the transcriptome of individual cells. The assembled data allow us to generate a catalogue of cell types, which are present in the selected tissue. Single-cell transcriptomics can, for example, make predictions, which cell type in the heart is most strongly affected by a given mutation or in a particular disease. The cellular resolution helps to gain further insight into the role of cell-cell interactions and cell type–specific effects of any disease process including CHD. *Fluorescence in situ hybridization* This technique makes use of chromosomal-specific DNA probes, which are labelled with a fluorochrome to recognize specific chromosomes, and in addition uses then specific DNA probes that hybridize with specific chromosomal regions, which, for example, are typically deleted or amplified in diseases such as a trisomy or the 22q11 deletion syndrome. With multiple chromosome-specific probes, the extent of a deletion in the affected patient can precisely be defined by this cytogenetic technique. *Multiplex ligation-dependent probe amplification (MLPA)* This genetic method uses several gene-specific primers to amplify a specific subset of genomic DNA to assess the presence of deletions or amplifications in a particular region of the genomic DNA. It is normally used to detect any CNVs, which could be a deletion or an amplification of a specific segment of DNA and are also found in patients with CHD. *Chromosomal microarray analysis (CMA)* Similar to MLPA, CMA is used for the detection of CNVs such as DNA repeats or microdeletions. Here the patient’s genomic DNA and a healthy control DNA sample are fluorescence labelled and hybridized to a DNA chip containing chromosomal DNA. Any deviation in the copy number will become visible by an enhancement or decrease of the fluorescent signal of the patient’s DNA relative to the control sample. *Genome-wide association studies (GWAS)* In order to associate gene variants with a specific disease, an observational study of a genome-wide set of genetic variants in different individuals is performed to see if any variant is associated with a particular trait such as hypoplastic left heart syndrome. GWAS typically focus on associations between single nucleotide variations or polymorphisms and traits such as major human diseases.

### Gene panel testing

Focused GPT applies next-generation sequencing (NGS) to sequence genes targeted by hybridization probe capture or polymerase chain reaction amplification, allowing a rapid and cost-effective analysis of a patient’s genome.^144^ Virtual gene panels have also been developed where all genes are sequenced by WES or WGS, but only those included in the virtual panel are analysed. GPT has been adopted to identify variants in disease-associated genes in specific subsets of CHD.^145^ As per current clinical guidelines, GPT is recommended for analysis of 15 gene variants (Table 2). Variants of these genes have been linked to several unique conditions such as aortic valve disease, ASD, VSD, TOF, HLHS, and 22q deletion syndrome.^145^ This approach focuses on the number of variants being analysed, allowing for deeper sequencing than WGS or WES and increased sensitivity for mosaicism. More focused and thus more manageable, streamlined analysis of a limited number of variants reduces the turnaround time from test to diagnosis, results in the identification of fewer VUS, and is relatively cost-effective compared with WES/WGS.^146^, ^147^, ^148^, ^149^

**TABLE 2:** Genes assessed in the congenital heart disease comprehensive panel

Gene name	Genomic locus	Function	Disease association
CHD7	8q12.2	Helicase	CHARGE disorder (OMIM: #214800)
ELN	7q11.23	Extracellular matrix protein	Supravalvar aortic stenosis (OMIM: #185500)
GATA4	8p23.1	Cardiac transcription factor	ASD 2 (OMIM: #607941) AVSD4 (OMIM: #614430) TOF (OMIM: #187500) VSD1 (OMIM: #614429)
GATA6	18q11.2	Cardiac transcription factor	ASD 5 (OMIM: #614474) ASD 9 (OMIM: #614475) Conotruncal heart malformations (OMIM: #217095) TOF (OMIM: #187500)
GDF1	19p13.11	Signalling protein	Congenital heart defects 6 (OMIM: #613854) Right atrial isomerism (OMIM: #208530)
JAG1	20p12.2	Signalling ligand	Alagille syndrome (OMIM: 188450) TOF (OMIM: #187500)
NKX2-5	5q35.1	Cardiac transcription factor	ASD 7 (OMIM: #108900) Conotruncal heart malformations (OMIM: #217095) HLHS2 (OMIM: #614435) TOF (OMIM: #187500) VSD3 (OMIM: #614432)
NKX2-6	8p21.2	Cardiac transcription factor	Conotruncal heart malformations (OMIM: #217015)
NOTCH1	9q34.3	Signalling receptor	Aortic valve disease (OMIM: #109730)
NOTCH2	1p12	Signalling receptor	Alagille syndrome (OMIM: #610205)
NR2F2	15q26.2	Nuclear receptor	Congenital heart defects 4 (OMIM: #615779)
TAB2	6q25.1	MAP kinase	Congenital heart defects 2 (OMIM: #614980)
TBX1	22q11.21	Cardiac transcription factor	22q deletion syndrome (OMIM: #192430, #188400) Conotruncal heart malformations (OMIM: #217095) TOF (OMIM: #187500)
TBX5	12q24.21	Cardiac transcription factor	Holt-Oram syndrome (OMIM: #142900)
TBX20	7p14.2	Cardiac transcription factor (T-box family)	ASD4 (OMIM: #611363)

Online Mendelian Inheritance in Man (OMIM) database (https://www.omim.org) #xxxxxx points to specific articles in OMIM with detailed information for each genetic condition.

ASD, atrial septal defect; AVSD, atrioventricular septal defects; CHARGE, coloboma, heart defects, atresia choanae, growth retardation, genital and ear abnormalities; HLHS, hypoplastic left heart syndrome; MAP, mitogen-activated protein; NOTCH1, neurogenic locus notch homolog protein 1; TOF, tetralogy of Fallot; VSD, ventricular septal defect.

Several studies have explored the diagnostic utility of gene panels in both prenatal and postnatal testing to provide a more accurate prognostic prediction in individuals with an increased predisposition to developing cardiac defects and to aid genetic counselling in families with a history of inherited CHD.^150^, ^151^, ^152^ Prenatal testing aims to determine the likelihood of the fetus having CHD and is usually prompted by abnormal screening test results or a family history of CHD. Hu et al.^150^ used targeted GPT in fetuses with either nonsyndromic or syndromic CHD, which had normal karyotypes and negative CMA results. The study revealed a 15.9% detection rate for pathogenic (including likely) variants, none of which were detected in the parents.^151^ Therefore, focused NGS testing can be employed to detect DNVs prenatally in fetuses of a suspected monogenic disorder. Postnatal testing aims to identify the cause of CHD in affected individuals and can help inform families about CHD risk in future pregnancies. Targeted GPT identified the likely causative variant in roughly a quarter to half of the families analysed.^151^^,^^152^ A caveat with focused GPT testing is the confined limit of assessing genetic variants in previously identified candidate genes rather than identifying any novel CHD gene.^150^ Moreover, there are significant differences in SNPs between ethnicities; hence, these mandate designing genome-wide population studies with a proportional ethnic representation.^153^

### Whole-genome sequencing and whole-exome sequencing

#### Whole-genome sequencing

Diagnostic WGS originally focused on disease-associated variants, analysed with a gene panel approach. Data processing limits and uncertainty surrounding the biological significance of noncoding variants limited its clinical utility to rare diseases or GPT approaches. However, WGS sequences the entire genome and can identify single-nucleotide variants, insertions, deletions, CNVs, and structural variations in a nonfocused manner.^8^ As discussed previously, using WES/WGS has contributed significantly to identifying genetic variants and candidate genes in CHD. Morton et al.^154^ identified a 1.3-fold increased cancer risk associated with genetic variations implicated in CHD; patients with CHD and extracardiac anomalies or neurodevelopmental delays were identified as having an even higher risk of developing cancer.

Recent advancements improved the diagnostic utility and speed of WGS. Rapid WGS maximizes automation of bioinformatic analysis to provide a genetic diagnosis within 50 hours. In critically ill infants with CHD, rapid WGS increased the molecular diagnostic rate to 46% compared with microarray and GPT (10%).^143^ Alankarage et al.^155^ adopted a 2-tier combined system of high-confidence gene screening and comprehensive analysis to improve the identification of clinically actionable variants in 97 families with probands born with CHD. Using a 2-tiered approach, an additional 9% of variants were identified (increased to 31%).

Compared with GPT approaches, WGS carries a much higher associated cost and results in the generation of much higher volumes of data, which must be processed and securely stored.^156^ However, as costs continue to fall, from an estimated US\$500 million to $1 billion per genome in 2003 to approximately $600 at the time of writing, its use and utility as a diagnostic and prognostic tool is only likely to increase.^156^

#### Whole-exome sequencing

Comparatively, WES selectively targets and sequences exons containing disease-causing variants, enabling the efficient identification of genetic variants implicated in CHD. As mentioned earlier, CHD presents with complex phenotypes; hence, WES provides a comprehensive and high-throughput approach to genetic analysis.

WES enables simultaneous sequencing of thousands of exons, allowing for the detection of single nucleotide variants, small indels, and structural variants that may contribute to CHD pathogenesis. For instance, WES carried out on 829 patients with TOF identified more than 30 genetic changes encompassing LOF variants, missense variants, and indels.^140^ Hence, clinicians and researchers can use WES data to locate specific mutations responsible for the patient’s phenotypic presentation, facilitating precise diagnosis and risk stratification.

In addition, WES can identify pathogenic variants that may be passed from affected individuals to their offspring, which is vital for genetic counselling and family planning. A prospective cohort study of trio-based WES in 197 fetuses found increased diagnostic yield of CHD compared with CMA and conventional karyotyping, especially in CHD cases associated with extracardiac abnormalities (14.7% in CHD with extracardiac abnormalities vs 11.5% in isolated CHD).^135^ A systematic review also found increased diagnostic yield from WES in CHD with extracardiac abnormalities (49%) and in cardiac shunt lesions (41%).^135^ This information can help families understand the recurrence risk of CHD in subsequent generations and provide options for prenatal diagnosis where relevant, especially for specific CHD cases, for example, those with extracardiac abnormalities. Moreover, by analysing large patient cohorts, researchers can identify recurrent genetic variants or novel genes not previously implicated in CHD.

Although WES has greatly advanced our understanding of the genetic basis of CHD, challenges remain. WES only focuses on exons and does not provide information about introns that comprise most of the genome and is important in understanding gene susceptibility and gene expression regulation. The genetic heterogeneity of CHD demands a large sample size and collaboration across research institutes to identify a complete spectrum of causative variants. However, WES is still relatively expensive compared with other techniques, thus limiting its accessibility for large-scale studies and clinical applications. Also, WES is primarily designed to detect single nucleotide variants and small indels; hence, it is less effective in detecting large structural variants such as CNVs, inversions, and translocations. Despite being able to detect these genetic variants using WES, understanding their functional significance is challenging as many are of unknown significance and differentiating between benign and pathogenic variants can be complex.

Both WGS and WES hold clinical relevance. When a patient presents with CHD and the underlying cause remains unclear or if the patient presents with a complex presentation, WGS/WES can potentially pinpoint the underlying pathogenic genetic variants responsible for the condition. In addition, these tools hold therapeutic potential; in some cases, specific genetic variants implicated in CHD can inform treatment options and guide surgical interventions. By identifying these pathogenic variants, clinicians can better comprehend the long-term prognostic information for patients and associated comorbidities. WGS and WES also facilitate clinicians in providing genetic counselling to patients and helping them make informed decisions about family planning.

## Translating Genomics Into Clinical Practice

**Figure 2:**
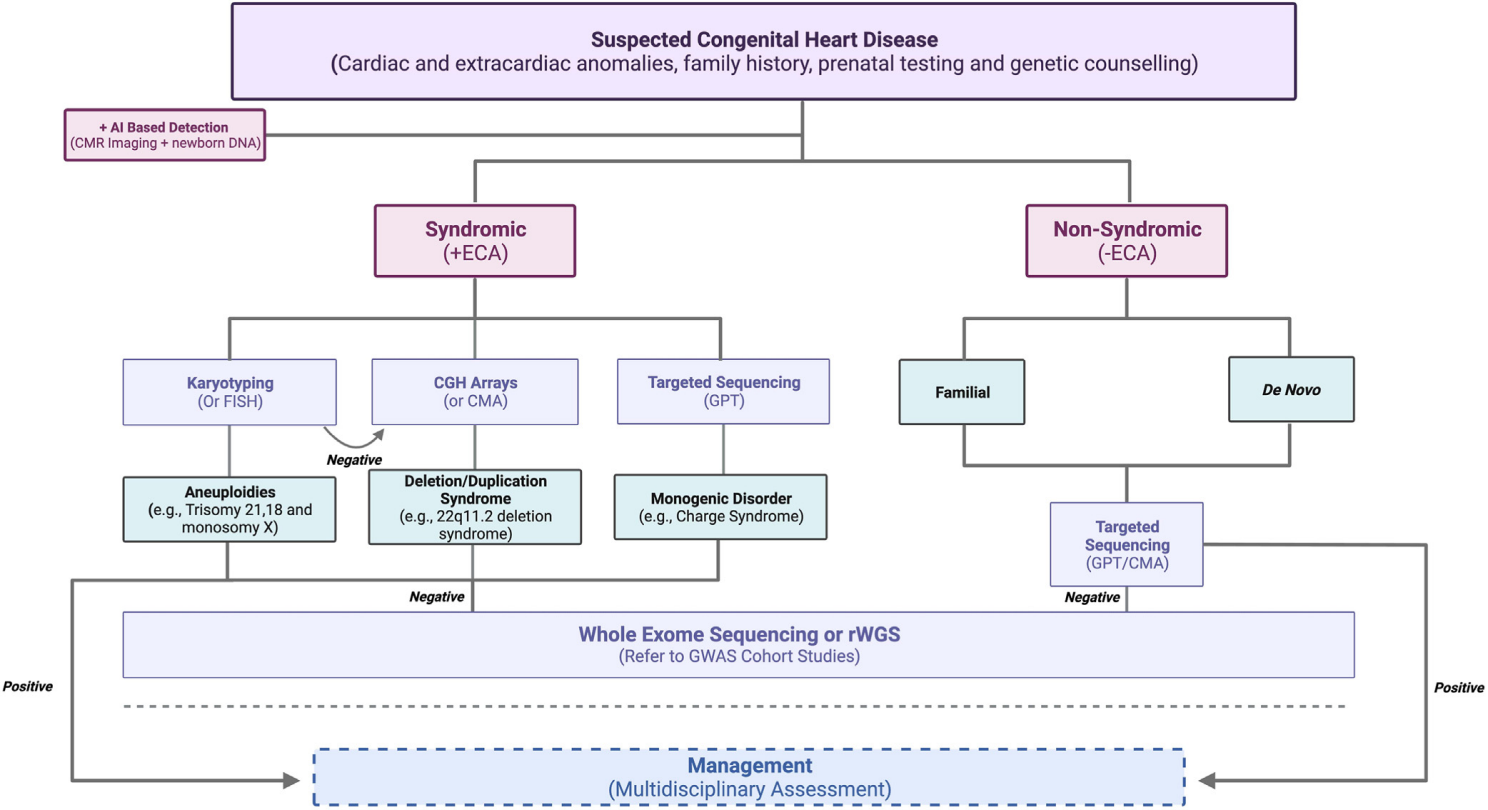
Framework for genetic testing in congenital heart disease. Artificial intelligence (AI)-based algorithms integrating cardiac magnetic resonance (CMR) imaging and neonatal DNA testing (newborn blood DNA analysis) can be adopted to risk stratify and accurately diagnose patients. *Syndromic pathway*: Extracardiac anomalies (ECA) are present. In suspected aneuploidies, karyotyping is performed, and if inconclusive, fluorescence *in situ* hybridization (FISH) is indicated. Subsequent negative results can be investigated with comparative genomic hybridization or chromosomal microarray analysis (CMA). The former 2 tests are the primary choice in suspected deletion/duplication syndromes, whereas targeted sequencing (gene panel testing [GPT]) is indicated in monogenic disorders. *Nonsyndromic pathway*: In the absence of ECA, irrespective of familial or *de novo* etiologies, targeted sequencing is the primary technique to assess for the underlying genetic aberration. Patients with negative results should be further investigated using whole-exome sequencing or rapid whole-genome sequencing (rWGS), in which novel pathogenic variants should be catalogued and referred to genome-wide association studies (GWAS) to substantiate the database of causative genetic variations within the congenital heart disease population. A positive result should prompt assessment by a multidisciplinary team on selecting the most appropriate targeted management to improve prognosis. This figure has been created using BioRender.

Clinical incorporation of clinical genetic testing is accelerating, partly due to reducing costs and increasing accessibility to genome sequencing. The earliest application of sequencing technologies to the clinic has been focused on syndromic CHD or CHD with known monogenic inheritance, such as GATA4 mutations, which are now known to be associated with septal defects.^157^ WES provides a comprehensive analysis of variants determining the inheritance of CHD and provides clinicians with a rich source of genetic information to risk profile patients. Successful clinical integration of genomic techniques depends on the appropriate use of genomic techniques in cases with a high pretest probability and informed consent. Therefore, it is necessary to define a clinical framework for appropriate genetic testing and subsequent utilization of these results (Fig. 2). The following section will detail the applications and considerations for the use of genomics in CHD clinics.

### Genetic testing and clinical prediction

Approximately 13.6% of individuals with CHD have associated extracardiac structural abnormalities. Many of these features are associated with a genetic syndrome, prompting genetic testing. However, more subtle variants may predispose groups to associated complications. Therefore, genetic testing may aid in risk stratifying individuals with CHD, enabling pre-emptive management of expected comorbidities and driving better predictive tools to assess the risk of postoperative complications and mortality.

In 223 patients with single ventricle disease, CMA identified CNVs >300 kilobases in 13.9% of patients and correlated adverse effects in growth and neurodevelopmental outcomes with these CNVs.^158^ CNV length has been shown to be negatively associated with postsurgical outcomes in chromosomal aneuploidies; hence, clinically, quantification of CNV length can be used as a novel tool in risk stratification.^34^^,^^51^ As large gene-overlapping novel CNVs in patients with CHD were significantly associated with significantly decreased transplant-free survival after surgery, CNV length could be an additional factor to consider when risk profiling patients for transplantation.^50^

Sequencing can assess patients with heterotaxy for primary ciliary dyskinesia, a predictor of postoperative mortality.^159^^,^^160^ Similarly, MYH6 variants in patients with HLHS have been attributed to worsening the prognosis for the clinical outcome and decreasing transplant-free survival.^161^^,^^162^ Furthermore, NGS can be used to establish databases of CHD gene variations, similar to PCGC, which can be used to build predictive algorithms to guide management.^51^

### Diagnostic advancements and responsible testing

As with any new diagnostic tool, great care must be taken to ensure the responsible incorporation of genomic testing in CHD into clinic. The currently high cost of genetic testing and the significant expertise required for the analysis and interpretation of NGS data are a barrier and potential polarizer of inequality in health care provision. Testing should be broadly available and carefully selected according to the needs of each patient. Care must be taken to ensure that focused, hypothesis-guided tests are performed where additional information is actionable and informs the development of a more personalized management plan. Clinicians should prioritize the “first do no harm” approach and ensure that patients are informed of the risks vs benefits of genetic testing.

As the cost of WGS and WES continues to fall, it may seem tempting to employ genome-wide untargeted sequencing; however, clinicians should carefully consider the implications of extended screening. Inappropriate use of extended screening may lead to difficulty interpreting VUS, incidental findings of known or unknown significance, and may even increase the risk of diagnostic error.^163^

For this reason and the limited number of published guidelines over the best approaches to adopt, the use of gene panels is increasing in CHD. However, there is little consensus on validated causal CHD genes. Griffin et al.^164^ studied the variability in gene panels across 3 different laboratories and found 16% congruency in genes assessed by all 3 laboratories. The same authors applied a Clingen framework to select 99 target genes of 558 genes identified by the PCGC for GPT, and diagnostic yields from CNV and WES were 1.8% and 3.8%, respectively. Continued efforts using data from the PCGC and the evidence from experimental models that aim to establish the biological significance of variants should help to inform the development of gene panels and increase consensus across centres. For this purpose, the results of the Congenital Heart Disease Curation Expert Panel are eagerly awaited.^165^

### Family planning

An identification of a genetic cause of CHD may be beneficial to both the individuals and their families. A genetic diagnosis may help increase confidence in the diagnosis, alert clinicians to screen for associated extracardiac abnormalities, and inform family planning as increasing numbers of individuals with CHD reach a child-bearing age.^166^ Guided by expert genetic or reproductive counsellors, individuals can make informed decisions about the risks of passing variants to their offspring. A comprehensive consensus statement from the European Society of Cardiology Working Group of Grown-Up Congenital Heart Disease gives valuable guidance regarding the timing of reproductive counselling and the risk of prenatal diagnostic tests.^167^ This may allow the patient access to services that provide guidance on choices of conception, adoption, or the consideration of reproductive techniques such as preimplantation genetic testing.

### Managing variants of unknown significance

Given the heterogeneous nature of CHD and the complex genotype-phenotype relationship, unbiased (WGS) may uncover disease-associated variants lacking a clear causal pathway to the phenotype. The interpretation of VUS can be challenging, and patients must be aware of the possibility of VUS discovery when consented to genetic testing. The implications of incidental findings (variants that confer a known predisposition to the development of a disease that is unrelated to the disease that prompted the test) unrelated to the cardiac structural abnormality discovered during WGS/WES must also be considered. A multidisciplinary approach, including geneticists and counsellors, is recommended to facilitate the interpretation of NGS results. Discovery of a new variant may prompt evaluation of the variant’s frequency in the population and disease-specific databases, which can indicate the likely pathogenicity of the variants based on its prevalence. In addition, employing *in vitro*, *in vivo*, and *in silico* models aids in determining the variant’s impact on cardiac morphogenesis, as outlined in the following section.

### Artificial intelligence

WGS and WES have generated large quantities of data, but limitations in data processing make establishing causal links difficult. AI is yet to be incorporated into large-scale CHD genome profiling cohort studies. AI-imaging algorithms were developed using cardiac magnetic resonance images from 372 patients with TOF to establish a risk-stratification algorithm to predict prognosis and complications such as ventricular tachycardia.^168^ Other algorithms have determined the feasibility and surgical risk associations and predict medication effects in CHD patients.^169^, ^170^, ^171^ Bahado-Singh et al.^172^ used AI to analyse causative epigenomic changes in newborn blood DNA and predicted nonsyndromic coarctation of the aorta in 24 patients. Genetic-based AI algorithms can be used clinically to predict prognosis in high-risk patients and diagnose patients earlier. Furthermore, AI can quantify the role of gene regulatory networks, developing accurate algorithms for predicting CHD phenotype (Fig. 2). Currently, there is poor standardization across clinical systems and a lack of comparable testing sets, a limitation that needs to be addressed.^173^

## Conclusion

Genomic technology has transformed our understanding of CHD and associated gene-environment interaction; thus, clinicians can now consider complexities of CHD cases. However, determining the exact effect of each epigenetic modification, coding, and noncoding variant remains a significant challenge. The effect of single variants can be determined using *in vitro* and *in vivo* models and epigenetic sequencing. AI advancements will improve WGS/WES data processing and lead to increasingly accurate prognostic prediction tools. However, large cohort sizes are needed, and recruitment is a significant barrier to increasing study power. Clinicians should promote patient uptake for these studies. Moreover, clinicians should become advocates for the integration of genomics into clinics and familiarize themselves with advancements in the field. Increasingly specialized multidisciplinary teams comprising cardiologists, researchers, geneticists, genetic counsellors, specialist care nurses, and social workers will be required to guide patients through genetic testing and the interpretation of these results to form personalized management plans.
